# Staging laparoscopy for locally advanced gastric cancer in Chinese patients: a multicenter prospective registry study

**DOI:** 10.1186/s12885-017-3791-6

**Published:** 2018-01-10

**Authors:** Ziyu Li, Zhemin Li, Lianhai Zhang, Qian Liu, Zhenjun Wang, Zhongtao Zhang, Gang Xiao, Wei Fu, Xin Wang, Yingjiang Ye, Jianchun Yu, Fei Li, Lin Chen, Shibin Wang, Jiafu Ji

**Affiliations:** 1 Key laboratory of Carcinogenesis and Translational Research (Ministry of Education), Department of Gastrointestinal Surgery, Peking University Cancer Hospital and Institute, Beijing, 100142 China; 2 Cancer Hospital and Institute, Chinese Academy of Medical Sciences, Beijing, 100021 China; 3 Department of General Surgery, Capital Medical University Affiliated Beijing Chao-Yang Hospital, Beijing, 100020 China; 4 Department of General Surgery, Beijing Friendship Hospital, Capital Medical University, Beijing, 100050 China; 5 Department of General Surgery, Beijing Hospital of Ministry of Health, Beijing, 100730 China; 6 Department of General Surgery, Peking University Third Hospital, Beijing, 100191 China; 7 Department of General Surgery, Peking University First Hospital, Beijing, 100034 China; 8 Department of Gastrointestinal Surgery, Peking University People’s Hospital, Beijing, 100044 China; 9 Department of General Surgery, Peking Union Medical College Hospital, Peking Union Medical College, Beijing, 100730 China; 10 Department of General Surgery, Xuanwu Hospital, Capital Medical University, Beijing, 100053 China; 11 Department of General Surgery, The General Hospital of PLA, Beijing, 100853 China; 12 Department of General Surgery, The First Affiliated Hospital, General Hospital of PLA, Beijing, 100048 China; 13 Key laboratory of Carcinogenesis and Translational Research (Ministry of Education), Department of Gastrointestinal Surgery, Peking University Cancer Hospital and Institute, Beijing, 100142 China

**Keywords:** Gastric cancer, Laparoscopy, Peritoneal metastasis, Cytology

## Abstract

**Background:**

Staging laparoscopy(SL) is a recommended technique for the staging of Gastric Cancer(GC) and provides the indication for a radical surgery. Considering the medical practice in China, the standardized and regular usage of SL is yet to be spread. However, existing guidelines vary and make an ambiguity of indication for SL. Besides, the specific indication for Chinese patients remains a niche. This study aims to the essential, missing information of Chinese patients and tries to normalize the indication of LS in medical practice in China.

**Methods:**

The study is a prospective, multicenter cohort study being conducted in China with a total of 450 patients, all diagnosed with locally advanced gastric cancer (cT2-4 N0-3 M0, no evidence of intra-abdominal dissemination) through Computed Tomography(CT) and/or Endoscopic Ultrasonography(EUS). Peritoneal lavage is regularly performed during the SL. Multivariate Cox regression model and receiver-operator characteristic(ROC) analysis will be used to analyze the significant risk factors of intra-abdominal metastasis(including peritoneal dissemination and a positive cytological result).

**Discussion:**

This confirmatory study will provide us with the specific positive rate of intraabdominal metastasis of GC in China, compared with empirical evidence of 20%. We expect this trial will contribute to our discovery of the specific risk factors of intra-abdominal metastasis of Chinese patients and to the stimulating and performing of minimally invasive surgical procedures.

**Trial registration:**

ClinicalTrials.gov: registration number NCT02172690.

## Background

Gastric cancer is a major cause of cancer death worldwide with a 40 percentage of cases occurring in China [1]. Individualized treatment based upon tumor stage takes on a widely accepted strategy, and surgery remains the most effective treatment modality.

Most of the Chinese patients are diagnosed with locally advanced stage, suffering the substantial intra-abdominal metastases with higher risks. Precise preoperative staging is particularly important for therapeutic strategies and prognostic evaluation.

Precise staging is a foremost method that benefits the successful surgical intervention of cancer, contributed to the improvement of medical tools: despite the fact that preoperative imaging techniques have significantly improved the diagnostic accuracy of wall infiltration and lymph node involvement, the detection of peritoneal dissemination still needs to be developed; positive intra-peritoneal findings of free cancer cells has been proved to be related with poor survival yet could only be confirmed during surgical procedure [2, 3].

SL has been suggested useful in detecting unrecognized peritoneal dissemination, and in collecting peritoneal lavage fluid for cytological examination with minimal invasion. However, indication for SL remains controversial. NCCN guideline recommends an indication for SL of T3 and/or N+; ESMO, IB-III; JGCA, none, merely mentioned SL only on the condition of neoadjuvant Chemoradiotherapy [4–6]. No specific recommended indications for Chinese patients can be referred to for lack of solid evidence.

The aim of this study is to acquire a basic data on the positive rate of SL in Chinese patients and to identify the risk factors associated with intra-abdominal metastasis.

## Methods

### The design of study

This study evaluates the efficacy of SL with peritoneal cytology, and determine the indications if possible.

**Fig. 1:**
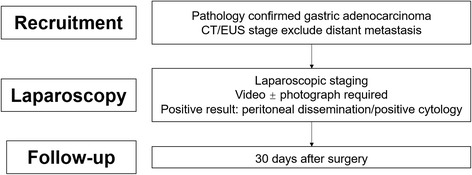
CONSORT diagram of study

Patients who meet the inclusion criteria will be enrolled consecutively, with their preliminary data collected using a web-based case report form. When the whole data-collecting is completed, factors associated with intra-abdominal metastasis will be analyzed by multivariate Cox regression model. Receiver-operator characteristic (ROC) will be used to find out risk factors of intra-abdominal metastasis. Figure 1 shows the CONSORT diagram.

#### Recruitment

Patients with pathologically confirmed gastric adenocarcinoma are recruited. If the patient meets all inclusion criteria, following data will be recorded: basic information including age, gender, height, body weight et al., clinical information including CT stage(enhanced CT is mandatory required), EUS stage, result of blood examinations such as tumor marker (CEA, CA19-9, CA72-4, CA12-5), blood cell count, albumin et al. If there been any differences between CT & EUS, it is required to record them separately.

#### Staging laparoscopy

If the patient is confirmed at stage cT2-4bN0-3 M0, which means no sign of distant metastasis detected by CT/EUS, he/she undergoes SL. Following data will be recorded: intraoperative finding including the peritoneal dissemination, and the peritoneal lavage; video of surgery and pictures demonstrating the extent of peritoneal dissemination is required.

#### Follow-up

Postoperative hospital stay and complications will be recorded, the follow-up time will be 30 days after surgery. Only those who undergoes DL alone will be included into safety analysis since the complications will be confused if the patient undergo radical surgery immediately after SL.

#### Study endpoint

The primary target of this study is to obtain the result of SL. Either peritoneal dissemination or a positive result of cytological examination will be recorded as a positive result. If the pathological or cytological examination describes “suspicious”, the result will be characterized as positive, consistent with our current practice.

The secondary aim is to evaluate the safety of SL.

### Selection of candidate patients

1. Age ≥ 18;
2. Histological confirmed diagnosis of primary gastric carcinoma (including esophagus-gastric junction).
3. Karnofsky score ≥ 70;
4. Abdominally enhanced spinal CT and endoscopic ultrasonography (EUS) showed a clinical stage of T2-4bN0-3bM0;
5. Fit for radical gastrectomy or neo-adjuvant chemotherapy;
6. Willing to comply with protocol, and provide informed consent forum.

The exclusion criteria included:

1. Unfit for laparoscopic operation or general anesthesia (suspicious abdominal adhesions, severe cardiopulmonary disease, etc.);
2. Underwent emergency surgery to relieve obstruction, bleeding or perforation.

### SL technique

SL was performed under general anesthesia. The patient is placed in a supine position. A 10-mm disposable trocar (observing hole) is inserted into the sub-umbilicus, and a 30° telescope is used. Another 10-mm trocar and a 5-mm trocar (operating hole) are inserted into the right upper quadrant and left upper quadrant, respectively. Prior to any manipulation, 250 ml of warm normal saline is infused into subphrenic space, subhepatic space, omentum, bilateral paracolic sulci and the pouch of Douglas. The irrigation should be carefully operated to avoid the primary tumor. At least 100 ml of fluid is aspirated from the subphrenic space, subhepatic space and Douglas’ pouch. The fluid is immediately sent for centrifugation and cytological examination and a systematic inspection of the abdominal cavity is performed clockwise from the right quadrant. Any suspicious lesion will be biopsied and sent for frozen pathologic examination.

### Sample size

This study is a single arm OPC (objective performance criteria) design where the primary evaluation index is the positive rate of SL. Based upon literature, expected event rate is 20%, and clinical acceptable rate is above 15%. Type I error as bilateral 5%, dropout rate as 5%, power as 80%, calculated sample size is 450. The recruitment process is estimated to be completed by December 2016.

### Data analysis

Clinical characteristics of the DL- and DL+ patients are compared using the Independent samples group t test and chi-square test. To determine the risk factors, all variables found to be significant by the univariate analysis are assessed by binary logistic regression analysis (Method: Backward: Wald, probability for stepwise: 0.5 for entry, 1.0 for removal). The ROC curve and AUC analyses are used to determine sensitivity, specificity, and corresponding cut-off value of each factor. All *P* values < .05 are considered statistically significant. All statistical analyses are carried out using the SPSS statistical software package (version 20).

## Discussion

This study is conducted to prospectively evaluate the positive rate of the result of SL based on samples of Chinese patient and try to discover risk factors of intra-abdominal metastasis. SL has been recommended by many guidelines yet different indications is suggested. The standardized operational procedure remains controversial for such practice along with possibly unknown bias. Druing the process of information collecting, data will be prospectively recorded using web-based case report forms.

The results of the study will provide the first prospective multi-center database of SL in China, according to our knowledge, which will provide important figure of 40% of patients of the world.

## Abbreviations

AUC: Area under curve
CT: Computed Tomography
EUS: Endoscopic ultrasonography
OPC: Objective performance criteria
ROC: Receiver-operator characteristic
SL: Staging laparoscopy
